# GLTP Is a Potential Prognostic Biomarker and Correlates with Immunotherapy Efficacy in Cervical Cancer

**DOI:** 10.1155/2022/9109365

**Published:** 2022-07-07

**Authors:** Yan-long Shi, Ming-bo Liu, Hong-ting Wu, Ye Han, Xuan He

**Affiliations:** ^1^Department of Medical, Women and Children's Hospital of Chongqing Medical University, Chongqing, China; ^2^Department of Obstetrics and Gynecology, Women and Children's Hospital of Chongqing Medical University, Chongqing, China; ^3^Cancer Center, Daping Hospital, Army Medical University, Chongqing, China

## Abstract

Cervical cancer (CC) is the fourth most commonly diagnosed cancer in women worldwide. The prognosis of CC patients remains poor. The objective of our study was to explore the potential of glycolipid transfer protein (GLTP) in predicting the prognosis of CC and patients' response to immunotherapy. The expression of GLTP was determined using TCGA and GEO datasets. The prognostic value of GLTP in CC patients was analyzed using Kaplan-Meier analysis and multivariate analysis. The relationships between BTBD10 and immunological checkpoints, immune checkpoint genes, and ferroptosis-related genes were analyzed to explore the impact of GLTP on CC immunotherapy. According to the dysregulated expressions of BTBD10, the IC50 distribution of various targeted medicines was studied. In this study, we found that GLTP expression was distinctly upregulated in CC specimens. However, Kaplan-Meier assays showed that CC patients with low GLTP expressions tended to exhibit a shorter overall survival. Importantly, multivariate assays revealed that GLTP expression was an independent prognostic factor for CC patients. Moreover, we observed that GLTP expression was related to CD4+ T cells, macrophages, and dendritic cells (DCs). Meanwhile, GLTP expressions were associated with those of immune checkpoints, ferroptosis-related genes, and m6A-related genes. The IC50 of Cisplatin, Docetaxel, and Paclitaxel was lower in the high-GLTP-expressing group. Taken together, GLTP was expected to be a prognostic and immunotherapeutic marker for CC.

## 1. Introduction

In recent years, cervical cancer (CC) has risen to the status of a major public health concern, being one of the most common forms of cancer in women globally [1]. Low-income countries and regions have 18-fold higher mortality rates than high-income countries, with underdeveloped countries accounting for 85% of all deaths [2]. With respect to its pathological pathways, human papillomavirus (HPV) is a major cause, and the dysregulation of genes is also an important factor [3, 4]. Although the application of HPV vaccine can prevent CC, more research into CC's therapeutic options and clinical outcome is urgently required [5, 6]. Thus, it is urgent to identify predictive biomarkers and therapeutic targets for treatments of CC and to study the mechanisms that underlie the occurrences and metastasis of CC.

Human glycolipid transfer protein (GLTP), located on chromosome 12 (locus 12q24.11), is a small (24kD) amphitropic protein. It has been confirmed that they are involved in the nonvesicular trafficking of various glycosphingolipids. In addition, their potential functions drug resistance, differentiation, neurodegeneration, surface adhesion, and apoptosis were also reported [7, 8]. GLTP possesses a unique all *α*-helical conformation, arranged in a two layer “sandwich motif” that forms a single “pocket-like” glycolipid binding site [9]. In recent years, several studies have reported that GLTP exhibited a dysregulated level in several types of tumors, such as colorectal cancer, lung cancer, and glioblastoma [10–12]. However, its potential tumor-related function and clinical significance in tumors remained largely unclear. In this study, our group aimed to explore the roles of GLTP in CC, including its expressions and prognostic values, tumor-related effects, and connection with the immune microenvironment.

## 2. Materials and Methods

### 2.1. Data Acquisition and Preprocessing

Clinical data from the CESC cohorts, as well as RNA-seq datasets (FPKM profiles), were collected from The Cancer Genome Atlas (TCGA) datasets. Then, a Perl script based on the CMD command was used to create the gene expression matrix. The Ensembl database was used to transform identifiers into gene symbols. Meanwhile, the external validation group included mRNA expression profiles (GSE44001 and GSE9750) that were retrieved from the Gene Expression Omnibus (GEO) database and used in the R affyPLM package's Robust Multiarray Average (RMA) technique. GSE44001 included 300 CC samples with recurrence (*n* = 38, recurrent; *n* = 262, nonrecurrent). GSE9750 includes data from 33 CESC samples and 24 normal samples. Predicting GLTP expression differences and correlations with tumor stage, grade, mutations in TP53, and methylation of DNA was done by using the UALCAN software [13].

### 2.2. Survival Analysis

The Youden index ((sensitivity + specificity) 1) was used to calculate the appropriate GLTP cut-off value, and the CC samples were separated into two groups: GLTP expressions range from high to low. Using the Kaplan-Meier (K-M) method and the log-rank test, we were able to compare and contrast the survival differences between our two groups. A *p* < 0.05 was regarded as statistically significant.

### 2.3. Immunoassay

The TIMER and TCGA datasets were utilized to investigate the connection between GLTP expressions and tumor infiltrating lymphocytes (TILs) [14]. GLTP and immunological checkpoints were examined in several groups to learn more about how GLTP affects TILs. The Tumor Immune Dysfunction and Exclusion (TIDE) algorithm was used to predict if ICIs will have a positive effect on the tumor. As part of a clinical immunotherapy trial, a Wilcoxon test was performed to examine the correlation between GLTP expressions and the expression of ferroptosis-related and m6A-related genes.

### 2.4. Inhibitory Concentration (IC50) Scores

The half-maximal IC50 is a critical marker for assessing a drug's efficacy or a sample's response to treatment. It is possible to forecast the efficacy of targeted and/or immunotherapy for CC by the use of Genomics of Drug Sensitivity in Cancer (GDSC).

### 2.5. Statistical Analysis

All statistical analyses were carried out using R 3.6.1 software. *p* < 0.05 was regarded as statistically significant.

## 3. Results

### 3.1. Identification of GLTP Expression in CC Specimens

**Figure 1:**
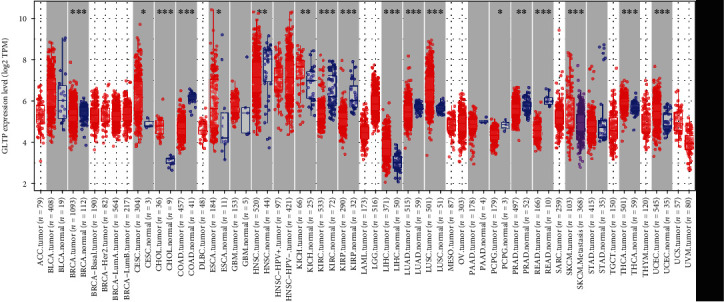
The TIMER analysis was used to determine the GLTP expression levels in diverse tumor specimens. GLTP exhibited a dysregulated level in many types of tumors. ^∗^*p* < 0.05, ^∗∗^*p* < 0.01, and ^∗∗∗^*p* < 0.001.

**Figure 2:**
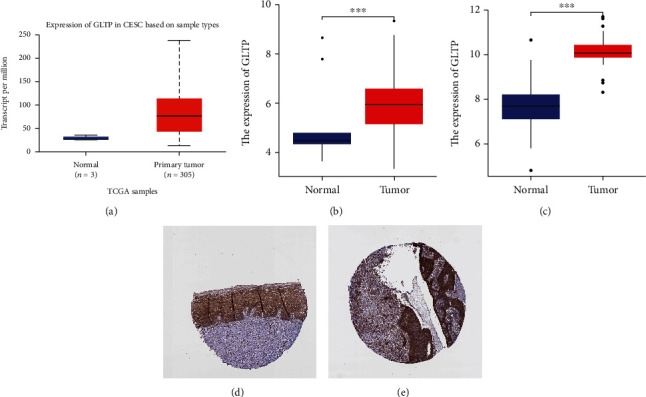
The expressing pattern of GLTP in CC and nontumor specimens. (a) UALCAN datasets; (b) TCGA datasets; (c) GSE9750 datasets. Representative images of immunohistochemical staining for GLTP in (d) nontumor specimens and (e) tumor specimens. ^∗∗∗^*p* < 0.001.

TIMER results predicted that GLTP expression in 10 types of tumor specimens (including CC specimens) was considerably greater than that in nontumor specimens (Figure 1). Meanwhile, our findings were supported by the data from the UALCAN datasets, the TCGA database, and the GSE9750 datasets (Figures 2(a)–2(c)). Further evidence of increased GLTP protein expression in cervical cancer tissues came from the Human Protein Atlas (HPA) immunohistochemical data as well (Figures 2(d) and 2(e)).

### 3.2. The Prognostic Value of GLTP Expression in CC Patients

**Figure 3:**
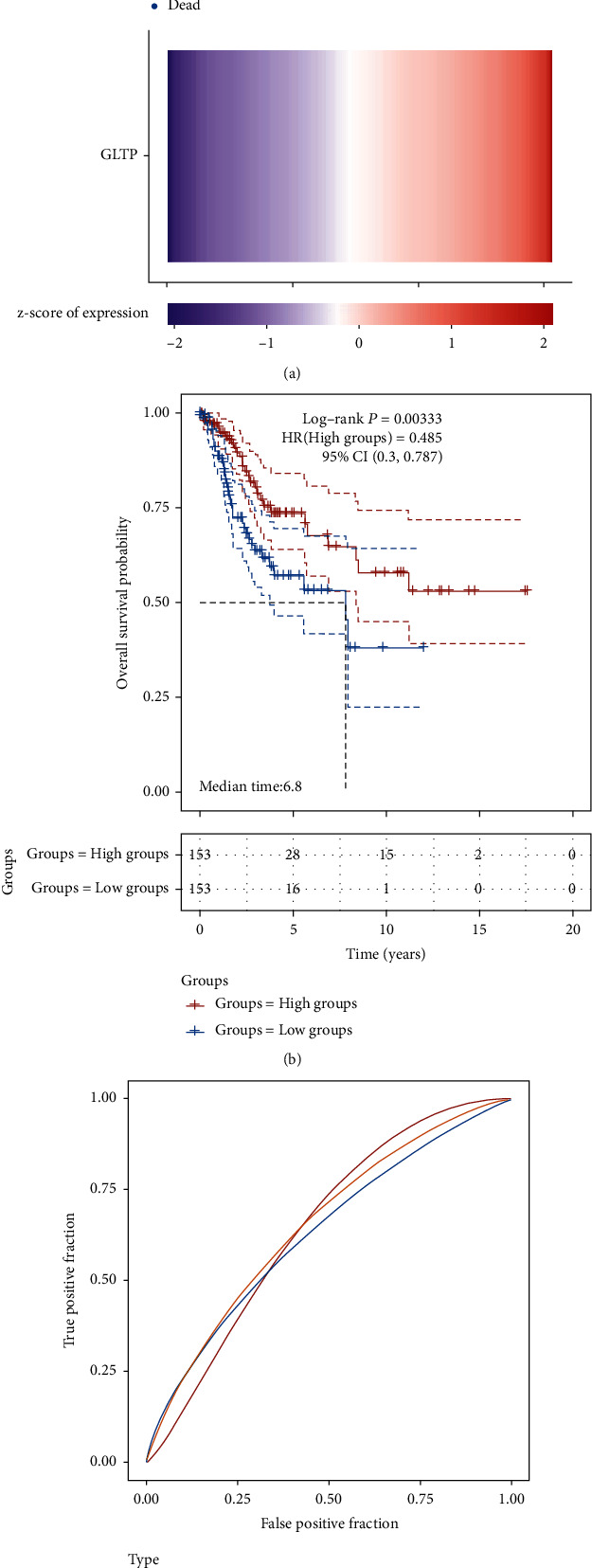
The prognostic value of GLTP in CC patients. (a) The survival status of CC patients from TCGA datasets. (b) Survival assays of all CC patients divided into two groups (high and low) based on the mean expression of GLTP. (c) ROC analysis of 1, 3, and 5 years. (d) LOW expression of GLTP was associated with poor prognosis of CC patients based on GSE44001 datasets.

**Figure 4:**
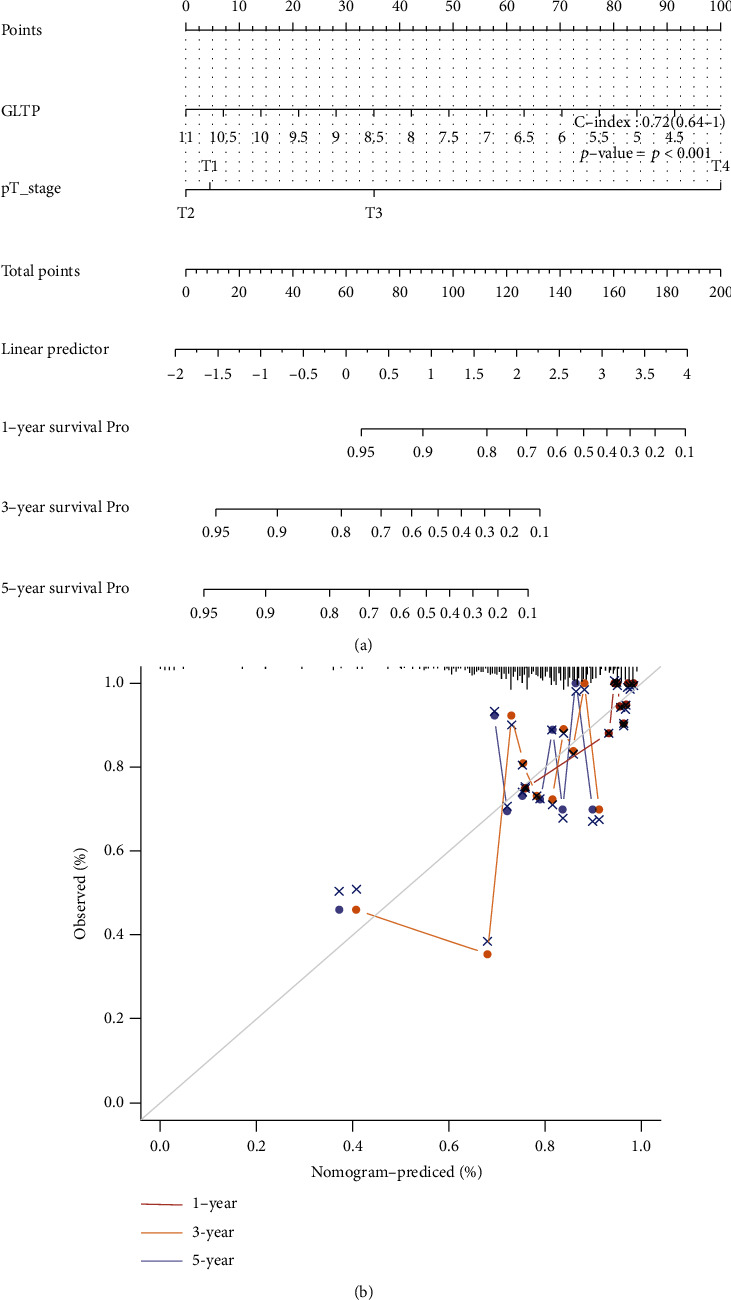
(a) A nomogram for predicting the overall survival of CC patients after one, three, and five years is shown. (b) The ideal nomogram calibration curve is depicted by a dashed diagonal line on the graph.

To explore the clinical significance of GLTP expression in CC patients, we analyzed TCGA datasets; the survival status of all CC patients is shown in Figure 3(a). In addition, patients with low GLTP expression showed a shorter overall survival than those with high GLTP expressions (Figure 3(b)). The ROC assays for 1, 3, and 5 years were performed, and the results are exhibited in Figure 3(c) (AUC > 0.6). On the other hand, we analyzed GSE44001 and also found that high GLTP expression was associated with a shorter overall survival (Figure 3(d)). GLTP was found to be an independent prognostic risk factor for CC patients in univariate and multivariate assays (Tables 1 and 2). An independent prognostic risk element, GLTP, provided a quantitative method for the prediction of the likelihood of overall survival at 1, 3, and 5 years for CC patients (Figures 4(a) and 4(b)).

### 3.3. GLTP mRNA Levels Are Associated with TILs

**Figure 5:**
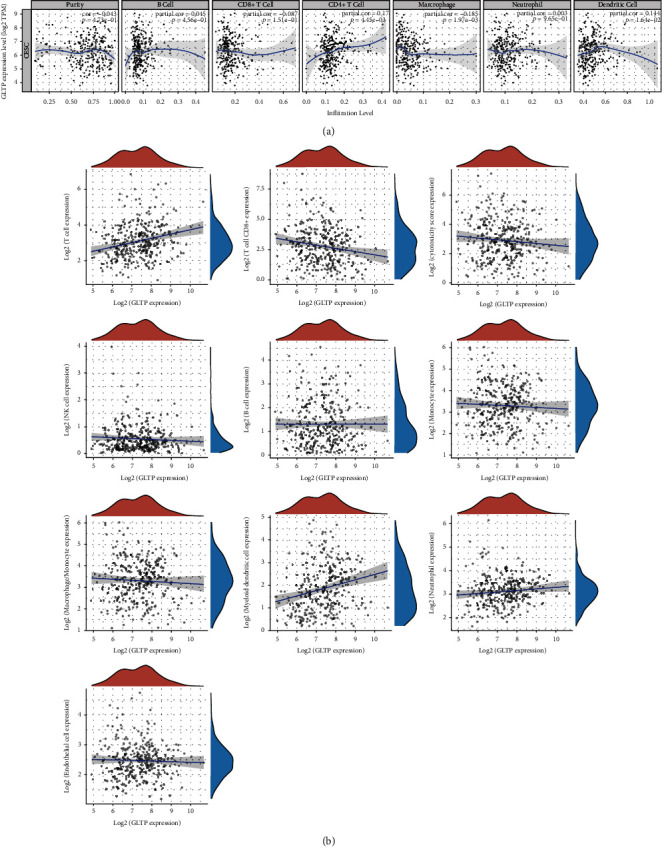
The associations between GLTP expressions and levels of infiltrating immune cells in CC. (a) TIMER website; (b) TCGA datasets from the ACLBI website.

Immune cells and TILs that expressed GLTP have been linked to a better prognosis in patients with CC. Cancer cells infiltrate the lymphocyte-specific immune recruitment (LYM) metagene signature, which is associated with better prognosis in CC. Therefore, we tested whether GLTP expressions in CC was related to TILs. TIMER and TCGA assays indicated that GLTP expressions were related to CD4+ T cells, macrophages, and dendritic cells (DCs) (Figures 5(a) and 5(b)).

### 3.4. Immunotherapy

**Figure 6:**
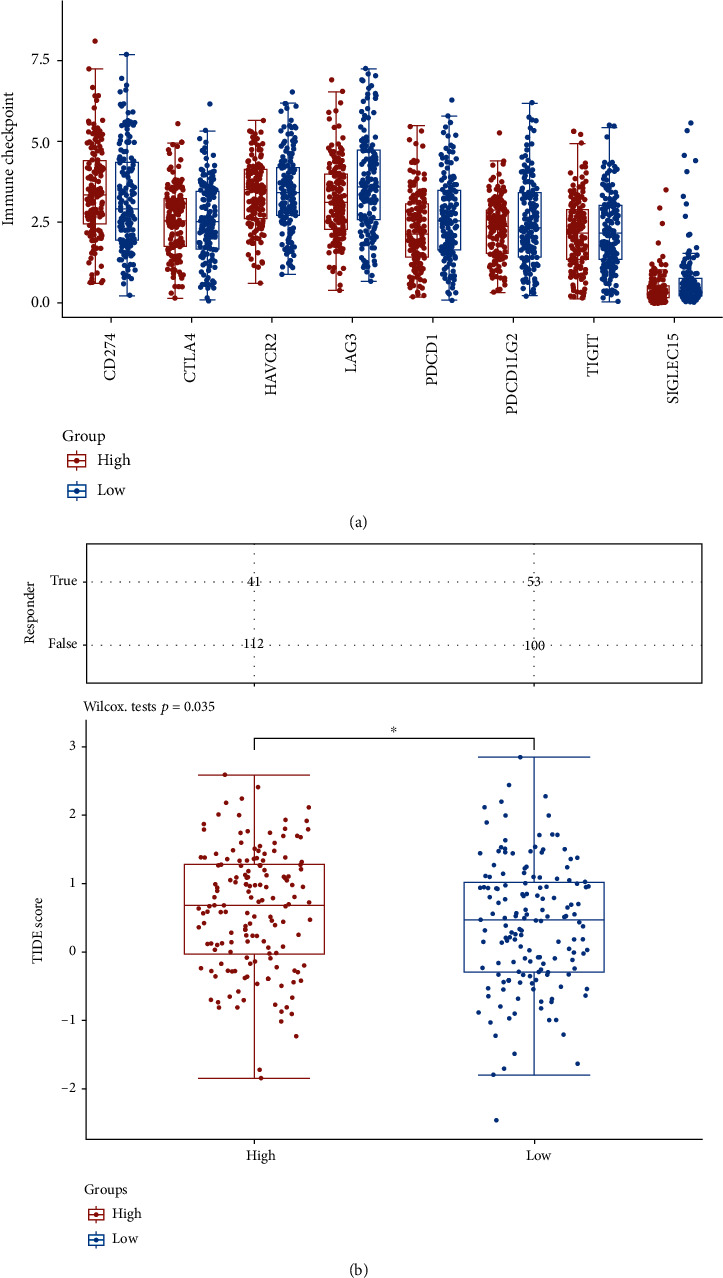
Expression distribution of immune checkpoint genes in two groups (high and low) based on GLTP expressions. (a) The expressions of immune checkpoints in the low GLTP expression group and high GLTP expression group. (b) For the statistical analysis of the immunological response, the TIDE method was employed. ^∗^*p* < 0.05, ^∗∗^*p* < 0.01, and ^∗∗∗^*p* < 0.001.

The surgical excision of advanced CC patients has gradually been substituted with immunotherapy. CD274 expression levels were considerably greater in the high-GLTP-expressing group than in the low-GLTP-expressing group in the TCGA datasets. However, LAG3, PDCD1, and SIGLEC15 expression levels were considerably greater in the low-GLTP-expressing group than in the high-GLTP-expressing group (Figure 6(a)). Subsequently, high-GLTP expression groups had poorer outcomes when treated with ICIs than low-GLTP expression groups, as demonstrated by TIDE (high TIDE scores, poorer response to ICIs, and short survivals after ICI treatments) (Figure 6(b)).

### 3.5. Connections Assays between GLTP and m6A Methylation

**Figure 7:**
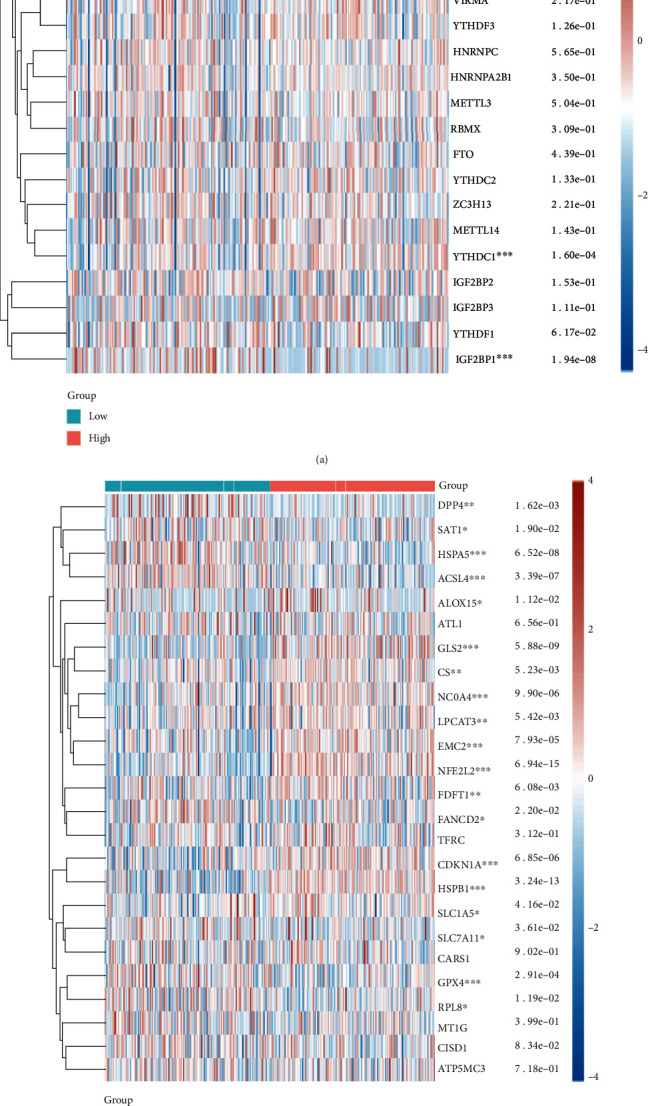
(a) Heat map of m6A methylation-associated gene expressions in two groups by the use of TCGA datasets. (b) Heat map of ferroptosis-associated gene expressions in two groups by the use of TCGA datasets. ^∗^*p* < 0.05, ^∗∗^*p* < 0.01, and ^∗∗∗^*p* < 0.001.

Antitumor immunotherapy has incorporated m6A methylation more frequently in recent years. Our group observed that GLTP expressions were related to m6A methylation-related genes, including RBM15B, YTHDF2, YTHDC1, and IGF2BP1 (Figure 7(a)). In light of this association, it is possible to improve the efficacy of targeted medications.

### 3.6. Connections Assays between GLTP and Ferroptosis

Antitumor immunotherapy can be made more effective by increasing ferroptosis in tumor cells [15, 16]. We observed that BTBD10 is correlated with DPP4, SAT1, HSPA5, ACSL4, ALOX15, GLS2, CS, NCOA4, LPCAT3, EMC2, NFE2L2, FDFT1, FANCD2, CDKN1A, HSPB1, SLC1A5, SLC7A11, GPX4, and RPL8 (Figure 7(b)).

### 3.7. IC50 Score

**Figure 8:**
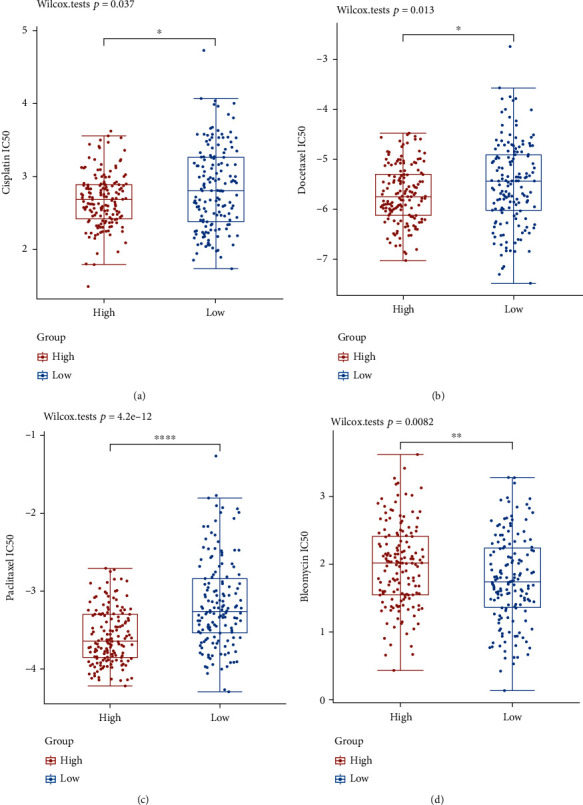
The difference of IC50 scores of targeted drugs in two groups (high and low) based on GLTP expressions via the ACLBI website: (a) Cisplatin, (b) Docetaxel, (c) Paclitaxel, and (d) Bleomycin. ^∗^*p* < 0.05, ^∗∗^*p* < 0.01, ^∗∗∗^*p* < 0.001, and ^∗∗∗∗^*p* < 0.0001.

A patient's response to targeted medication therapy can be gauged by their IC50. We used GDSC data to forecast the differences between GLTP expression groups in IC50 scores of chemotherapeutic drugs. The IC50 of Cisplatin, Docetaxel, and Paclitaxel was lower in the high-GLTP-expressing group, while the IC50 of Bleomycin exhibited a higher trend (Figures 8(a)–8(d)).

## 4. Discussion

Over 300,000 women die each year as a result of CC, one of the world's worst diseases [17]. There is still a terrible prognosis for patients with CC, despite breakthroughs in prevention, diagnosis, and treatment [18, 19]. Thus, understanding the molecular mechanism of CC's development is so critical. In recent years, several studies have reported that GLTP was dysregulated in several types of tumors [10, 20, 21]. However, the studies on GLTP in CC were rarely reported. Based on clinical data from TCGA and GEO datasets, we observed that GLTP was highly expressed in CC. Interestingly, we observed that patients with low GLTP expressions were related to poor outcomes of CC patients from both TCGA and GSE44001 datasets. Importantly, univariate and multivariable Cox regression analysis identified low GLTP expression in CC tissues as an independent poor prognostic marker of overall survival. According to our studies, GLTP can accurately forecast the dotted line, which was found to be very accurate. These findings suggest that GLTP could be a valuable marker for CC prognosis and diagnosis.

Nonmalignant cells and inflammatory substances produced by stroma and tumor cells can influence tumor growth in the tumor microenvironment (TME) [22]. There is a hurdle to immunotherapy because of the TME's immune-suppressive properties [23]. In the context of a complex microenvironment, multiple immune cells coexist and interact, leading to the failures of antitumor immune surveillance [24, 25]. For instance, T-cell activation is inhibited by chemokines produced by tumor-associated macrophages, which attract Tregs [26]. Furthermore, BMP4 activates cancer-associated fibroblasts, which inactivate NK cells and allow tumor cells to evade the immune system [27]. Targeting immune-suppressing cells is a hot topic of research. These investigations shed light on ways to improve immunotherapy. In this study, we analyzed TCGA datasets and found that GLTP expression was positively correlated with CD4+ T cells, macrophages, and dendritic cells. These results indicated that GLTP may have a role in the regulation of the immune system by interfacing with tumor-infiltrating lymphocytes and macrophages.

Prognostic markers for the disease have been established [28, 29]. However, treatment for CC is not ideal because drug response is usually a complex feature, often influenced by many genomic and environmental factors. Our results showed that patients with strong GLTP expression had a higher TIDE score than those with low GLTP expression (the higher the TIDE scores, the worse the efficacies of ICIs). For patients with advanced CC, targeted medications have been the standard of care, but the effectiveness of single therapies ranges from 12 to 20 percent, which limits their therapeutic applicability in practice [30, 31]. As a result, it is critical to improve the precision and personalization of immunotherapy treatment by better predicting and evaluating its success in clinical settings. Dual immune checkpoint blockage has been demonstrated to improve antitumor immunity to CC in studies. According to TIDE data, SAT1 expression was lower in the high-expression group of GLTP compared to the low-expression group, and our study found that the high-expression group's ICIs ORR was lower in comparison. Because of this, patients with low GLTP expressions may benefit from targeted drugs combined with ferroptosis-related therapies, whereas patients with high GLTP expressions may benefit from multiple targeted drug combinations or combined m6A-related therapies, both of which could be promising treatment options for those suffering from CC.

Although the possible roles of GLTP in the modulation of immune response in the progression of CC had been studied, more cellular experiments are necessary for the identification of the associations between GLTP expressions and CC progression and then to illustrate the specific mechanisms involved in GLTP expression in CC progression. In addition, it is a retrospective study, which means it has the usual selection and reporting biases. To corroborate our findings, further investigations on a large number of patients are needed due to the small sample size.

## 5. Conclusion

Our results provide evidence that GLTP expression was increased in CC. However, high expression of GLTP was associated with favorable prognosis. It is possible for GLTP to be considered a novel prognostic biomarker for CC. In addition, GIHCG may affect the infiltration and function of immune cells in CC. Our findings suggested the prognostic and therapeutic roles of GLTP in CC patients, providing potential therapeutic targets for CC patients.

## Figures and Tables

**Table 1:** Univariate and multivariate cox regression analyses of risk factors associated with overall survival based on TCGA datasets.

Characteristics	Total (*N*)	Univariate analysis		Multivariate analysis	
		Hazard ratio (95% CI)	*p* value	Hazard ratio (95% CI)	*p* value
Age	306				
≤50	188	Reference			
>50	118	1.289 (0.810-2.050)	0.284		
T stage	243				
T1 & T2	212	Reference			
T3 & T4	31	3.863 (2.072-7.201)	<0.001	5.924 (2.166-16.200)	<0.001
Clinical stage	299				
Stage I & stage II	231	Reference			
Stage III & stage IV	68	2.369 (1.457-3.854)	<0.001	0.749 (0.274-2.045)	0.573
GLTP	306				
Low	153	Reference			
High	153	0.550 (0.343-0.882)	0.013	0.455 (0.253-0.819)	0.009

**Table 2:** Univariate and multivariate cox regression analyses of risk factors associated with overall survival based on GSE44001 datasets.

Characteristics	Total (*N*)	Univariate analysis		Multivariate analysis	
		Hazard ratio (95% CI)	*p* value	Hazard ratio (95% CI)	*p* value
STAGE	300				
Stage I	258	Reference			
Stage II	42	1.325 (0.691-2.538)	0.397		
GLTP	300	0.626 (0.445-0.881)	0.007	0.626 (0.445-0.881)	0.007

## Data Availability

The original data supporting the conclusions of this paper will be provided unreservedly by the authors to any qualified researcher.
